# From Sweeping to the Caress: Similarities and Discrepancies between Human and Non-Human Primates’ Pleasant Touch

**DOI:** 10.3389/fpsyg.2016.01371

**Published:** 2016-09-08

**Authors:** Laura C. Grandi

**Affiliations:** Department of Neuroscience, University of ParmaParma, Italy

**Keywords:** non-human primates, pleasant touch, grooming, CT fibers, primates

## Abstract

Affective touch plays a key role in affiliative behavior, offering a mechanism for the formation and maintenance of social bonds among conspecifics, both in humans and non-human primates. Furthermore, it has been speculated that the CT fiber system is a specific coding channel for affiliative touch that occurs during skin-to-skin interactions with conspecifics. In humans, this touch is commonly referred to as the caress, and its correlation with the CT fiber system has been widely demonstrated. It has been hypothesized that the sweeping touch that occurs during grooming in non-human primates may modulate the CT fibers, with recent preliminary studies on rhesus monkeys supporting this hypothesis. The present mini-review proposes a comparison between the pleasant touch, caress and sweeping of humans and non-human primates, respectively. The currently available data was therefore reviewed regarding (i) the correlation between pleasant touch and CT fibers both in humans and non-human primates, (ii) the autonomic effects, (iii) the encoding at the central nervous system, (iv) the development from early life to adulthood, and (v) the potential applications of pleasant touch in the daily lives of both humans and non-human primates. Moreover, by considering both the similarities and discrepancies between the human caress and non-human primate sweeping, a possible evolutionary mechanism can be proposed that has developed from sweeping as a utilitarian action with affiliative meaning among monkeys, to the caress as a purely affective gesture associated with humans.

## The Touch in Primates

The sense of touch assumes a critical importance in daily life, since it enables not only the ability to discriminate and haptically explore and identify objects, with the optional integration of other sensory information (the sensory-discriminative aspect), but it also allows us to communicate with others while creating and maintaining social bonds according to the emotional valence that touch assumes (the motivational-affective aspect). The static touch responsible for the discriminative aspect activates the large myelinated low threshold mechanoreceptors (LTMRs) to allow the rapid encoding of an object’s features at the central nervous system level. Conversely, the affiliative touch activates the C tactile unmyelinated LTMRs (CT fibers), to instead allow the processing of the emotional meaning of the touch.

Since the role of touch has historically been considered to be discriminative, this dimension of touch has been well studied, with the affective aspects being recognized to a lesser degree and only recently being investigated, despite their importance in social interaction (Mountcastle, 2005).

These two components of touch are present among both humans and non-human primates. Indeed, all non-human primates utilize the discriminative aspect of touch to identify objects and food, while the emotional aspect determines the codification of the meaning of social interactions such as negative outcomes when in conflict and positive outcomes when grooming. The mechanisms of the discriminative component’s encoding in non-human primates are similar to those found in humans. Nevertheless, our current understanding of the mechanisms behind the codification of pleasant touch in non-human primates is limited. Recently, it was proposed that the role of CT fibers during the sweeping that occurs during allogrooming echoes that which manifests in the caress among humans (Dunbar, 2010). Despite it being accepted that the CT fibers are also present in the hairy side of non-human primates’ skin (Kumazawa and Perl, 1978), there are no studies exploring the correlation between CT fibers and pleasantness, as found in humans. The scope of this mini-review is thus to discuss the potential parallelism between the mechanisms behind the encoding of the caress among humans, and that of sweeping among non-human primates.

## The Role of CT Fibers in Human Affective Touch

CT fibers were first identified in the hairy skin of cats through the saphenous nerve preparation in 1939 (Zotterman, 1939), and then in various mammals including mice, rats, guinea-pigs, rabbits, pigs and non-human primates (Douglas and Ritchie, 1957; Bessou et al., 1971; Iggo and Kornhuber, 1977; Kumazawa and Perl, 1978). These fibers are activated by dynamic tactile stimulation manifesting on the hairy side of the skin at the specific speed of 1–10 cm/sec, while their firing rate decreases for velocities lower than 1 cm/sec and higher than 10 cm/sec. As a result of the unmyelination the conduction speed is low, specifically at 0.6–1.3 m/s (Kumazawa and Perl, 1978; Vallbo et al., 1995).

As these fibers were not detected in the hairy or glabrous skin of humans for a significant period of time, it was assumed that they had disappeared during the evolutionary process due to their physiological properties. For instance, the absence of myelin covering the axons determines the slow conduction velocity, which is not evolutionarily advantageous in terms of rapidly detecting the characteristics of a touched object. It was not until the late 1980s that Johansson et al. (1988) reported their presence for the first time in the infraorbital nerve of humans, followed by other studies which underscored that the hairy side of the arms and legs were indeed also innervated by unmyelinated CT fibers (Nordin, 1990; Vallbo et al., 1995).

Importantly, the numerous human studies conducted to understand the functional role of unmyelinated CT fibers underscored that the touch which activates these fibers is perceived as pleasant by those subjects who receive it (Löken et al., 2009). From an autonomic perspective, touch determines the positive physiological effects toward vagal modulation, such as the decrement of heart rate (HR) and increment of heart rate variability (HRV) (Field et al., 1986; Tsao, 2007; Garnera et al., 2008; Russell et al., 2008).

Moreover, a potential link has been posited between the pleasantness of slow, CT-optimal touch and opioid signaling. For instance, the administration of naloxone, an opiate antagonist with high affinity for the μ-opioid receptor, reduced the preference for CT-optimal touch in healthy subjects (Case et al., 2016); therefore, opioid withdrawal appears to alter the value of social touch (Loseth et al., 2014). The endogenous opioid system is believed to underpin the rewarding nature of social relationships, and may mediate the pleasantness and reward of CT-related social touch (Panksepp et al., 1980; Dunbar, 2010).

In respect to the central processing, the CT fibers project into the inner lamina I and II of the spinal dorsal horn (Light and Perl, 1979; Sugiura et al., 1986). Then, the information is relayed via the ventromedial posterior thalamic nucleus to the dorsal posterior insular cortex ([pIC]; Olausson et al., 2002, 2008a,b, 2010; Björnsdotter et al., 2009); the primary and secondary somatosensory areas (Gazzola et al., 2012); the orbitofrontal cortex; the postero-superior temporal sulcus; the medial prefrontal cortex; the dorso-anterior cingulate cortex; and the pregenual anterior cingulate cortex (Kringelbach and Rolls, 2004; McGlone et al., 2007, 2012; Gordon et al., 2013; Ellingsen et al., 2014). In particular, the pIC appears to have a pivotal role in the encoding of the pleasant touch mediated by the modulation of CT fibers (Olausson et al., 2008a,b; McGlone et al., 2014). This evidence is consistent with the notion that the CT fibers belong to the interoceptive system and play a pivotal role in the encoding of the pleasant component of interpersonal touch.

Based on these data, different theories have been proposed concerning the social role of CT fibers. For instance, Morrison et al. (2010) proposed the ‘skin as a social organ’ hypothesis, positing the role of the touch’s affective dimension by means of CT fiber modulation, in the transmission and processing of social information. Olausson et al. (2010) forwarded the ‘social touch’ (or ‘affective touch’) hypothesis, according to which the social touch is a distinct domain of touch that requires a specific pathway, i.e., the CT fiber system. Gordon et al. (2013) identified a network of *social brain* regions (Brothers, 1990) – a complex neural network specialized to support social function – that are implicated in the processing of CT-targeted touch, supporting the hypothesis that CT fibers serve a specific function in the social processing of touch.

Taken collectively, these findings from different approaches including (i) behavioral analysis related to the pleasantness of the stimulation, (ii) the autonomic effects, and (iii) brain imaging studies, support the notion that the CT system is a specific coding channel for gentle, dynamic touch occurring during close affiliative relationships among humans.

## Grooming as an Affiliative Behavior Among Non-Human Primates

Echoing the gentle caress for humans, allogrooming is a social affiliative behavior for non-human primates. Allogrooming is a widespread behavior, primarily carried out to clean others’ body parts that are inaccessible or invisible to self-grooming (Barton, 1985), and for the control of lice infection (Zamma, 2002). Nevertheless, the amount of time devoted to grooming exceeds that necessary for cleaning, suggesting that there is a social reason beyond the hygiene function (Kummer, 1968; Boccia et al., 1989; Spruijt et al., 1992). Indeed, allogrooming is the most common affiliative relationship and social strategy to create and maintain relationships and reliable alliances (Maestripieri, 1993; De Waal, 2008; Dunbar, 2010; McFarland and Majolo, 2011). It has been reported that allogrooming enhances relaxation and the sense of security (Dunbar, 2010), while simultaneously reducing anxiety levels (Schino et al., 1988; Boccia et al., 1989). These effects have received empirical support from studies investigating physiological parameters, such as the HR and cortisol levels. In particular, a decrement of the HR when receiving grooming (Boccia, 1989), and a reduction of the cortisol levels during both passive (Gust et al., 1993) and active grooming (Shutt et al., 2007) was shown. Moreover, it has been demonstrated that (i) allogrooming elicits endogenous μ-opioid release, (ii) the injection of μ-opioid agonists such as morphine determined a reduction in the engagement in social grooming, and (iii) the μ-opioid antagonists such as naloxone determined an increment in the tendency to receive grooming (Meller et al., 1980; Fabre-Nys et al., 1982; Keverne et al., 1989; Schino and Troisi, 1992). In addition to the numerous studies focused on allogrooming, the behavioral and physiological impact of grooming conducted by humans on monkeys has also been explored. It was demonstrated that the human grooming of rhesus monkeys is a positive reinforcement in operant conditioning (Taira and Rolls, 1996), determining the increment of HRV and decrement of HR, and thus resulting in autonomic modulation toward vagal tone activation (Grandi and Ishida, 2015).

## Human and Non-Human Primates’ Pleasant Touch: A Brief Overview of Ontogenesis and Importance From Early Life

Touch is the first sense to ontogenetically develop (Gallace and Spence, 2010). Somatosensory responses can be detected in utero as early as 8 weeks gestational age (Arabin et al., 1996; Bystrova, 2009). This suggests that fetal movement in the amniotic fluid stimulates CTs that activate the hypothalamus and insular cortex, thereby promoting an anti-stress effect through oxytocin release, as well as potentially providing the developing social brain with its primary template.

Throughout infancy and early childhood the sense of touch provides important information and feedback concerning the surrounding world, while influencing the development of the motor, social, and communication skills. Moreover, being touched decreased infants’ stress-activated cortisol production and increased cell development in the hippocampus, impacting on both the short- and long-term memory functions (Miles et al., 2006). In addition, when given with a velocity of 3 cm/sec the caress determined the decrement of HR of 9-month-old infants (Field, 2010). Furthermore, the parental affiliative tactile behavior during the early stages of neural development has a significant impact on the consequential behavior in adulthood (Hofer, 1995; Cascio, 2010; Suderman et al., 2012; Voos et al., 2013).

Affective touch is also considered to be crucial for non-human primates’ social and physiological development. In the milestone experiments of Harlow (1958) on maternal deprivation in rhesus monkeys, the importance of the affiliative relationship between infant and mother from the first days of birth was demonstrated, as well as the negative effects due to the absence of such interactions on the development and behavior of monkeys.

## Non-Human Primates Sweeping and Human Pleasant Touch: The Similarities

Grooming is characterized by bimanual actions with rhythmic sweeps and plucking movements (Tanaka, 1995). The sweeping is the dynamic hand action that the agent monkey performs to move the fur of the recipient monkey in order to expose the site of the skin to be picked for removal of the ectoparasites and vegetation trapped within. Due to the affiliative function of allogrooming (Dunbar, 1991, 2010; Kapsalis and Berman, 1996) and the characteristics of the sweeping motion, it is possible to consider allogrooming as the equivalent of social interpersonal human touch. In particular, the sweeping could be considered analogous to the human caress due to its own dynamic characteristic, as proposed by Morrison et al. (2010).

Nevertheless, while the sweeping is performed with the unique aim of moving the fur to clean it, the human caress is in contrast a purely affiliative gesture. According to Dunbar’s (2010) hypothesis of the CT fibers’ role in sweeping, this action does not solely have a hygienic function, but also involves affiliative meaning. Recent evidence supports this hypothesis. First, the mean speed of sweeping grooming touch among free ranging monkeys is 9.31 cm/sec, which is within the optimal velocity range (1–10 cm/sec) required to activate the CT fibers in humans. Moreover, the human sweep on the back of a male rhesus monkey performed with speeds of 5 and 10 cm/sec determined a decrement of the HR and increment of the HRV (Grandi et al., 2015). Similar autonomic effects have been demonstrated to be determined by the human caress, and it is well known that these cardio-physiological effects are indices of the positive modulation of vagal tone (Task Force of the European Society of Cardiology and the North American Society of Pacing Electrophysiology, 1996; Berntson et al., 1997; Eckberg, 1997). In addition, the human sweep on the back of a male rhesus monkey determined an increment of the nose skin temperature (Grandi and Heinzl, 2015), representing an index of the positive autonomic effects in the rhesus monkey (Nakayama et al., 2005; Kuraoka and Nakamura, 2011; Ioannou et al., 2015).

Moreover, a recent pilot study (Grandi and Gerbella, 2016) concerning the encoding of pleasant touch at the central nervous system level demonstrated the role of the insular cortex (pIC) in the encoding of pleasant touch in non-human primates at the single neuron level, a region that also appears to play a pivotal role in the encoding of pleasant touch in humans (see above). In Grandi and Gerbella’s (2016) study, it was shown that a population of pIC neurons encoded the received sweep with a speed of 5–15 cm/sec, in similarity to that which positively modulated the vagal tone of monkeys and comprising the speed with which rhesus monkeys performed this action in their natural environment (Grandi et al., 2015).

Furthermore, the endorphins play a central role in the motivational and social reasons for grooming, as human studies demonstrated a possible link between the pleasantness of slow, CT-optimal touch and opioid signaling.

This evidence indirectly supports Dunbar’s (2010) aforementioned hypothesis regarding the involvement of CT fibers during sweeping. In fact, it is performed at a similar velocity to the pleasant touch that activates CT fibers in humans (1–10 cm/sec), determines the same positive physiological effects in terms of HR (decrement) and HRV (increment), and involves endorphins and the modulation of the same brain regions (mainly the pIC). Furthermore, the effects of pleasant touch on the body temperature, as detected by means of infrared thermography, were reported for the first time in non-human primates (Grandi and Heinzl, 2015).

These data represent the first evidence of the representation of affiliative gentle sweeping at both the autonomic and central nervous systems in non-human primates, and highlight the similarity between the social touch systems of humans and non-human primates.

## Non-Human Primates Sweeping and Human Pleasant Touch: The Discrepancies

Beyond the above mentioned analogies, it is possible to underscore a number of discrepancies between the non-human primate and human studies related to pleasant touch. Indeed, the mean velocity of the real sweeping in non-human free ranging primates is 9.31 cm/sec, which is almost at the upper limit (10 cm/sec) of the range that activates human CT fibers. Moreover, the positive autonomic effects in terms of HR, HRV and nose skin temperature were obtained during human sweeping performed at velocities of 5 and 10 cm/sec, and therefore at the upper limit of the optimal range that activates the human CT fibers. Similarly, the pIC was selectively modulated during sweeping performed in the 5–15 cm/sec velocity range.

Therefore, human studies demonstrated that in order to activate CT fibers, determine the positive autonomic effects and modulate the insular cortex, the optimal velocity is 1–10 cm/sec; meanwhile, non-human primate studies suggested that the optimal velocity appears to be 5–15 cm/sec.

## Non-Human Primates Sweeping and Human Pleasant Touch: A Method To Improve Wellbeing

Human studies showed that the caress and moderate massages are utilized to enhance the wellbeing not only of people suffering from depression, chronic pain, stress, neurological or psychological disease, and for cancer patients receiving chemo- and radio-therapy, but also to reduce the stress experienced by healthy people (Belinda et al., 2008; Billhult et al., 2009; Diego and Field, 2009; Lindgren et al., 2010; Field, 2014; Schroeder et al., 2014).

Reinhardt and Reinhardt (2008) hypothesized that positive physical contact with personnel could be a method to enhance the welfare of single-house caged experimental non-human primates. Therefore, human grooming (Taira and Rolls, 1996; Grandi and Ishida, 2015) and human sweeping (Grandi and Heinzl, 2015; Grandi et al., 2015) could prove useful in reducing the stress under which experimental animals may find themselves during experimental conditions.

## Conclusion: From Non-Human Primates Sweeping to the Human Caress

Due to the importance of social touch, human studies suggest that evolution conserves a specific coding channel to allow its processing, i.e., the CT fiber system (Damasio and Carvalho, 2013; McGlone et al., 2014). In non-human primates, despite many studies hypothesizing on the social role of grooming, it was proposed that the sweeping which occurs during grooming could be considered homologuous to the human affective touch (Morrisson et al., 2010), and that rather than grooming *per se*, this motion may modulate the CT fibers (Dunbar, 2010). Moreover, recent preliminary data support this hypothesis, since similarities have been demonstrated between the human caress and non-human primate sweep.

Taken together, the aforementioned discrepancies and similarities between non-human primates sweeping and the human caress suggest that it is possible to speculate on an analogy between human and non-human primates’ CT systems as a coding channel for the processing of the affective touch.

Nevertheless, more detailed studies are necessary to deeply investigate the sweep among monkeys in order to more precisely determine the optimal velocity of the modulation of CT fibers, and to specifically demonstrate their activation during sweeping by means of direct measurement. Studies in this direction will confirm the homology between humans and non-human primates’ affective systems, as mediated by the CT fibers. The currently available data could offer an important starting point to explore the evolutionary mechanism behind the transformation of sweeping among non-human primates from a utilitarian action performed during grooming to clean others, to the caress as an affiliative gesture among humans.

## Author Contributions

The author confirms being the sole contributor of this work and approved it for publication.

## Conflict of Interest Statement

The author declares that the research was conducted in the absence of any commercial or financial relationships that could be construed as a potential conflict of interest.
